# Response to Editorial: Balancing the benefits of maternal nutritional interventions; time to put women first!

**DOI:** 10.1093/ajcn/nqz077

**Published:** 2019-08-01

**Authors:** Nancy F Krebs, K Michael Hambidge

**Affiliations:** From the Department of Pediatrics, Section of Nutrition, University of Colorado Anschutz Medical Campus, Aurora, CO

Dear Editor:

The editorial (1) accompanying our report of the Women First trial results (2) contained a number of factual misrepresentations. We are writing to clarify, in our view, the most important ones.

The first is the erroneous statement that the overall dropout rate exceeded 50%. As indicated in the CONSORT diagram, of the 7387 randomly assigned women, the dominant reasons for “trial exit” were either becoming pregnant <3 mo into the study or not becoming pregnant within the timeframe the study allowed to meet sample size goals. Only ∼30% (average for all arms) left the study during the preconception period due to moving out of the study area or no longer wishing to participate. Of the 44% of the randomly assigned women who conceived ≥3 mo after randomization and who entered the pregnancy phase of the trial (*n* = 3251), <3% for all arms exited the study. Of those with live births, the primary outcome was obtained for >90% of the participants. Preconception trials are inevitably faced with the inherent challenge of capturing enough pregnancies in the course of the study period to obtain outcomes. To provide relevant context, the percentage of conceptions of those randomly assigned for the Women First trial (44%) is favorably comparable to 2 other recently published preconception trials: the Mumbai Maternal Nutrition Project (3) and the PRECONCEPT trial in Vietnam (4). These trials followed 35% and 36% of randomized participants through pregnancy, respectively.

The second major misrepresentation in the editorial was "the choice of 3 mo as the timing of the preconception intervention,” surmising that this choice as a cutoff for preconception supplementation might have limited its impact. In fact, 3 mo was the *minimum* exposure; the actual average duration of exposure to the primary supplement for Arm 1 (preconception) was more than 9 mo. The rationale for that timeframe is available in the protocol article (5) but is also consistent with the other preconception trials, both of which also targeted a 3-mo minimum exposure to intervention (3, 4).

Finally, the comment questioning the value of providing a limited repertoire of micronutrients is puzzling because the primary lipid-based supplement contained >20 micronutrients, including 1000 IU of vitamin D.

We appreciate the opportunity to highlight these apparent misunderstandings in our study design and implementation. All of the details noted previously are included in the primary article for any readers who desire to review them directly.
